# Pathology of the Mouth—Excessive Formation of Epithelium

**Published:** 1860-06

**Authors:** 


### PATHOLOGY OF THE MOUTH—EXCESSIVE FORMATION OF EPITHELIUM.

Chronic excess in the quantity, and unusual tenacity of adhesion of the epithelial scales of the mouth, occurs in some persons, but this disease is not very common. Persons thus affected, even while in ordinary health, have a thick, yellowish coating upon the back part of the tongue. On the advent of any morbid derangement of the digestive apparatus, the coating covers the tongue quite to its tip, appears white, and uniformly distributed. It is a disease that is more frequently met with in those whose skin is inactive, with derangement or obstruction of the sebaceous folicles.

*Treatment.* Although this derangement does not produce much active distress, yet the *disease* that produces it demands treatment, which, however, consists in avoiding the producing causes, rather than in active medication. Smoking tobacco always increases this trouble, and the accompanying distress at the pit of the stomach. Opium, as well as tobacco smoking, produces this kind of furred tongue in well persons, and where the condition already exists neither tobacco nor opium can well be borne.

### ACUTE INFLAMMATION OF THE MOUTH.

Acute inflammation of the lining of the buccal cavity, when not produced by local agents, like similar acute inflammation in any other part of the system, seems always to be a result of a previous derangement of the fluids of the system.

The papillæ become swollen, prominent; do not shed their epithelium, which becomes harder and more tenacious than natural, and rising with the papillæ gives them the appearance of being short hairs, and the tongue of being *furred*, and not simply *coated* as where there is only an excess of epithelium.

When the tongue is *furred* it indicates that a febrile, or inflammatory change has occurred in the blood, and that the whole system is affected with a disease, for a cure of which *general* treatment will be required. The derangement of the alimentary canal may also be great, and require special attention. But a *furred* tongue always indicates a change in the fluids of the body.

*Treatment.* In the treatment of acute inflammation of the mouth, the derangement of the general system, and consequent demand for general treatment must never be lost sight of.

When the mucous membrane of the mouth and stomach is inflamed, there appears to be a difficulty in regard to the transmission of fluids through the membrane, and hence the system suffers from a deficiency of fluids and thirst of fevers is felt, often the sense of thirst leads the patient to drink copiously, but the fluid drank, not being absorbed from the stomach, passes into the intestines, and thence out, causing purging or a continuous diarrhœa. When the evacuations are produced by the non-absorption of the drinks, it is not proper to check them; for, in addition to relieving the stomach of its superabundance of liquid, it washes away the epithelium which has been shed as well as any other effete matter that has found lodgement in the alimentary canal. As in the treatment of Catarrh, so in this form of disease, the cautious and proper use of laxatives and diluents are beneficial and rational.

So, also, the use of agents that increase the amount of flow through the kidneys and the skin will not only lead to an increase of absorption from the mouth and stomach, but at the same time lessen the inflammation and tend to cleanse the tongue of its fur. Blood-letting will sometimes apparently cleanse the tongue; but if it produces any notable debility, bleeding causes the coating to become sticky, smooth, thick, and of a brownish color, and dryer. The stain produced by medicine or food upon the tongue *may* be mistaken for the changed condition here referred to; but with proper observation and thought such mistakes are not likely to occur.

Inflammation of the mucous lining of the mouth may occur in Catarrh, but such attacks are transient, and require no special treatment. Usually more attention must be paid to the general condition of the system that caused the local disease than to the mouth itself.

ANÆMIA OF THE MOUTH.

The Anæmic state of the lining of the mouth is more readily distinguishable by the appearance of the sub-epithelial blood-vessels than by the change it produces in the epithelium or the papillæ.

The first change noticed, may be that the tongue is somewhat swollen or tumid, and the papillæ appear enlarged. The epithelium appears nearly white as though the tongue was parboiled, thickened, soft, wrinkled, forming furrows, with a large number of epithelial scales floating in the saliva, giving it a whitish obaque appearance and an unpleasant taste. The breath has a peculiar unpleasant odor, doubtless also caused by the decomposition of some of the epithelial scales, a large amount of which are floating in the saliva or adherent to the teeth.

After the disease has progressed, the tongue becomes smoother and clean, the epithelum not having been formed as fast as washed away; the papillæ are flattened, the surface of the tongue quite smooth, the breath loses its unpleasant odor, the tongue becomes pale, flat, and so broad that the edges press against the teeth which make indentations in its border and its tips that remain quite a long time.

The anæmic state of the tongue always indicates a *general*, not local disease. Local diseases, even of the stomach, do not produce this anæmic condition of the mouth. Ulceration, and even cancer of the stomach or œsophagus do not lead to this condition: neither do derangements of the liver, the spleen, the kidneys, nor of more distant organs.

Sore throat, bronchitis, a decayed tooth, *may*, however, greatly modify the appearance of the mouth and tongue; so that it is always well to observe if there is any immediately adjoining disease that modifies the appearance of the tongue. So, also, if there is much disease of the tooth upon one side of the mouth as to cause the other one to be used exclusively in mastication, the epithelium not be worn away upon the other by the food, may give it the appearance of being diseased even in a state of health. The use of tobacco causes such a modification in the appearance of the mouth and tongue that its influence should never be lost sight of. So also does the habitual presence of many other things in the mouth; and it is well, always, to become acquainted with the habits and occupation of all whose mouths we are called upon to inspect.

